# Lead detour

**DOI:** 10.1007/s12471-019-01321-z

**Published:** 2019-08-12

**Authors:** L. M. van den Broek, S. W. Westra, R. Evertz, M. Boulaksil

**Affiliations:** Department of Cardiology, Radboud University Medical Centre, Nijmegen, The Netherlands

## Answer

In this case, an isolated persistent left superior vena cava (PLSVC) was present. A PLSVC is a remnant of the left cardinal vein which is normally obliterated during early embryological development, leaving the ligament of Marshall [1]. Failure of this regression will leave a PLSVC. In about 90% of cases, a PLSVC drains into the coronary sinus [2].

A PLSVC usually coexists with a right-sided superior vena cava (RSVC) or, less frequently, with a persistent bridging vein. In our case, however, a very rare isolated PLSVC was present, in which regression or obliteration of the RSVC occurs. Though rare, PLSVC is the most common venous anomaly, with a prevalence ranging from 0.3 to 0.5% in the general population and up to 10% in patients with congenital heart disease [2]. An isolated PLSVC is even rarer, with a prevalence of 0.07–0.13%. Usually, it is asymptomatic and an incidental finding during an implantation procedure or on imaging. A dilated coronary sinus on echocardiography should raise suspicion of a PLSVC.

In patients with an isolated PLSVC, especially right ventricular lead placement can be technically demanding due to the acute angle between the coronary sinus ostium and the tricuspid valve. In our case, we used a pigtail-shaped stylet to loop the lead in the right atrium and enter the right ventricle.

During our procedure, it became clear that advancement of the leads through the RSVC was impossible, so we decided to cross over to the PLSVC. Eventually, a right-sided dual-chamber pacemaker was successfully implanted. Left-sided device implantation through a PLSVC may be complex, but also remains a feasible approach. Long-term results of pacemaker implantation in patients with an isolated PLSVC are good [3]. In conclusion, we implanted a right-sided pacemaker with the leads through an isolated PLSVC.
